# Captopril alleviates hypertension-induced renal damage, inflammation, and NF-κB activation

**DOI:** 10.1590/1414-431X20187338

**Published:** 2018-09-03

**Authors:** Zhongyuan Gan, Dan Huang, Jiaye Jiang, Yuan Li, Hanqing Li, Yan Ke

**Affiliations:** Experimental Center for Teaching and Learning, Shanghai University of Traditional Chinese Medicine, Shanghai, China

**Keywords:** Captopril, Spontaneously hypertensive rats, Renal injury, Inflammation, Nuclear factor-κB

## Abstract

Hypertensive renal damage generally occurs during the middle and late stages of hypertension, which is typically characterized by proteinuria and renal inflammation. Captopril, an angiotensin-converting enzyme (ACE) inhibitor, has been widely used for therapy of arterial hypertension and cardiovascular diseases. However, the protective effects of captopril on hypertension-induced organ damage remain elusive. The present study was designed to explore the renoprotective action of captopril in spontaneously hypertensive rats (SHR). The 6-week-old male SHR and age-matched Wistar-Kyoto rats were randomized into long-term captopril-treated (34 mg/kg) and vehicle-treated groups. The results showed that in SHR there was obvious renal injury characterized by the increased levels of urine albumin, total protein, serum creatinine, blood urea nitrogen, renal inflammation manifested by the increased mRNA and protein expression of inflammatory factors including tumor necrosis factor-α, interleukin (IL)-1β, IL-6, and inducible nitric oxide synthase, and enhanced nuclear factor-κB (NF-κB) activation. Captopril treatment could lower blood pressure, improve renal injury, and suppress renal inflammation and NF-κB activation in SHR rats. In conclusion, captopril ameliorates renal injury and inflammation in SHR possibly via inactivation of NF-κB signaling.

## Introduction

Hypertension is a global chronic disease, and uncontrolled hypertension usually leads to chronic kidney disease and ultimately kidney failure (1). Hypertension-induced renal damage is a significant cause of morbidity and mortality in hypertensive patients and has become an important public health problem (2). The elucidation of mechanisms underlying hypertensive renal injury, as well as the development of new therapies to blunt its progression, are urgently required (3).

Angiotensin-converting enzyme (ACE) inhibitors are pharmaceutical drugs used primarily for the treatment of hypertension and congestive heart failure. In addition, ACE inhibitors have also been used in chronic kidney diseases (4). Captopril, an ACE inhibitor, has been demonstrated to exhibit protective effect on diabetic (5) and non-diabetic (6) renal injury. However, the possible protective action of captopril on hypertensive renal damage is poorly understood. This study was designed to investigate the effects of long-term treatment with captopril on hypertensive renal injury.

## Material and Methods

The 6-week-old male spontaneously hypertensive rats (SHR) and age-matched normotensive Wistar-Kyoto (WKY) rats were purchased from Shanghai Slack Laboratory Animal Co., Ltd (China). All the animals were housed in individual cages on a 12-h light-dark cycle in a room with controlled temperature (24±2°C) and humidity (50–60%), and allowed access to standard rodent chow and distilled water *ad libitum*. All experimental procedures were conducted in accordance with the National Institutes of Health Guide for the Care and Use of Laboratory Animals and were approved by the Ethics Committee of Shanghai University of Traditional Chinese Medicine (No. 2014025). After one week of accommodation to environmental conditions, the animals were treated with captopril (34 mg·kg^-1^·day^-1^, *po*; Bristol-Myers Squibb, USA) or vehicle (distilled water) for 5 weeks or 13 weeks. Thereafter, the rats were sacrificed and the left kidneys were carefully removed and sliced into two parts; one part was snap-frozen in liquid nitrogen and kept at –80°C for RNA and protein extraction, and the other part was immersed into 10% neutral-buffered formalin for histopathological examinations.

### Blood pressure measurement

Systolic blood pressure was measured every 2 weeks using a tail-cuff plethysmography (Alcott Biotech, China) as described previously (7).

### Measurement of renal function

After treatment with captopril or distilled water for 6 or 13 weeks, the rats were placed in metabolic cages. Urine samples were collected for 24 h while rats were fasted, but with free access to water. After the urine samples had been centrifuged at 1000 *g* for 10 min at room temperature, the total urinary albumin (ALB) and total protein (TP) were measured using kits from Fosun Long March Medical Science (China) according to the manufacturer’s instructions. The blood samples were taken from the abdominal aorta for determination of serum creatinine (CREA) and blood urea nitrogen (BUN). CREA assay kit and Quick Auto Neo BUN kit from Shino-Test Corporation (Japan) were used for serum CREA and BUN test according to the manufacturer's protocol, respectively.

### Histopathology examination

The fixed kidney tissues were embedded in paraffin and cut into 4-μm sections. Paraffin-embedded kidney slices were finally deparaffinized, rehydrated and stained with hematoxylin/eosin (HE) for microscopic study to assess histologic changes. The preparations obtained were visualized using a light microscope (Olympus, Japan) at a magnification of 200×. A pathologist examined each section in at least 10 randomly selected non-overlapping fields under a light microscope in a blind manner. The renal histopathology was quantified for the degree of Bowman’s space enlargement, tubular cell necrosis, exfoliated cells in the lumen, vascular congestion, and intratubular casts (8 [Bibr B09]). The level of each manifestation was graded according to the changes involved in comparison with the control group, scoring 0 for no changes, 1 for changes less than 20%, 2 for 20–40% of change, 3 for 40–60% of change, 4 for 60–80% of change, and 5 for greater than 80% of change compared to the control. Finally, the total histopathological score was calculated as the sum of scores for all regions of the nephrons.

### Quantitative real-time PCR

Total RNA was isolated from kidney cells using Trizol reagent (Invitrogen, USA) and reverse transcribed to complementary DNA (cDNA) using the RevertAid First Strand cDNA Synthesis Kit (Fermentas, China). The synthesized cDNA was amplified by a standard PCR protocol using SYBR green PCR master mix from Tiangen Biotech (China). The sequences of rat-specific primers for tumor necrosis factor-α (TNFα), interleukin (IL)-1β, IL-6, inducible nitric oxide synthase (iNOS), and GAPDH used in the study are listed in Table 1.

**Table 1.** Primers used for quantitative real-time PCR.

Genes	Forward primer	Reverse primer
*TNFα*	5′-GGAGAAACCTGCCAAGTATGA-3′	5′-TACCAGGGCTTGAGCTCA-3′
*IL-1β*	5′-CTCTCAAGCAGAGCACAG-3′	5′-TTCCATGGTGAAGTCAAC-3′
*IL-6*	5′-TACCCCAACTTCCAATGC-3′	5′-GATGGTCTTGGTCCTTAG-3′
*iNOS*	5′-ATCCCGAAACGCTACACTT-3′	5′-CGGCTGGACTTCTCACTC-3′
*GAPDH*	5′-GGAGAAACCTGCCAAGTATGA-3′	5′-CCCTGTTGCTGTAGCCATATT-3′

### Protein extraction and western blot

The renal tissues were lysed in a buffer (50 mM Tris at pH of 7.5, 1 mM EDTA, 150 mM NaCl, 20 mM NaF, 0.5% NP-40, 10% glycerol, 1% protease inhibitor cocktail and 1% phosphatase inhibitor) and homogenized on ice. The supernatant was collected after centrifugation at 13,000 *g* at 4°C for 20 min, and the protein concentration was determined by BCA protein assay kit (Pierce, USA).

Equal amounts of proteins (80 μg) were separated by 10% SDS polyacrylamide gel electrophoresis (SDS-PAGE) and transferred to a nitrocellulose membrane (Bio-Rad Laboratories, USA). After blocking, the membrane was incubated with primary antibodies at 4°C overnight. Antibodies used in the present study included: TNF-α (1:1000 dilution; 500-P72, PeproTech Inc., USA), NF-κB p65 (1:1000 dilution; 3034, Cell Signaling, USA), p-NF-κB p65 (1:1000 dilution; 3033S, Cell Signaling), IκBα (1:1000 dilution; 4812S, Cell Signaling), p-IκBα (1:1000 dilution; 2697S, Cell Signaling), p-IKK (1:1000 dilution; 2697P, Cell Signaling), iNOS (1:500 dilution; ab3523, Abcam, UK), IL-1β (1:1000 dilution; 500-P80, PeproTech Inc.), IL-6 (1:1000 dilution; 500-P73, PeproTech Inc.), and GAPDH (1:5000 dilution; ab8245, Abcam). After three washes with PBS-T buffer, the membranes were incubated with HRP-labeled goat anti-rabbit or anti-mouse IgG for 2 h at room temperature. The protein bands were detected using ECL reagents. Chemiluminescent signals were detected and analyzed using the ChemiDoc XRS Imaging System (Tanon, China).

### *In vitro* pharmacology

Rats were anesthetized, and the renal artery was excised and cut into rings of approximately 2 mm in length. The endothelium-dependent vasorelaxation was measured using the 620M myograph system (DMT, Denmark) as described previously (9). Briefly, rings were suspended in bicarbonate buffer solution at 37°C and continuously aerated with 95% O_2_, 5% CO_2_ for isometric tension recording in organ chambers. After a 60-min equilibration period under a resting tension of 1.5 g, the arteries were contracted with potassium-rich (60 mM K^+^) buffer solution with the same composition as the standard solution, except that NaCl was replaced by an equimolar concentration of KCl. Thereafter, the rings were contracted by phenylephrine (PE, 10^-6^M) and relaxed with a cumulative concentration of acetylcholine (ACh, 10^-9^∼10^-5^ M) to investigate the endothelium-dependent vasodilatation.

### Statistical analysis

Data are reported as means±SE, and n refers to the number of rats. Multiple group comparisons were performed using one-way ANOVA and LSD tests with SPSS18.0 (IBM, USA). The differences were considered statistically significant when P<0.05.

## Results

### Effect of captopril on body weight and blood pressure

**Figure 1.**
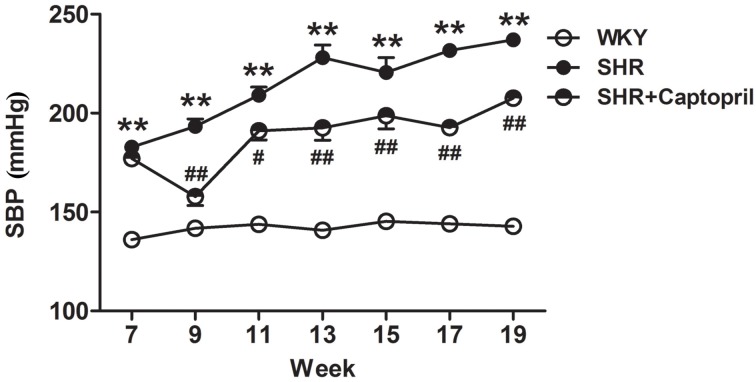
Time course of systolic blood pressure (SBP) of Wistar-Kyoto (WKY) and spontaneously hypertensive (SHR) rats measured by tail-cuff method. Data are reported as means±SE (n=6 in each group). **P<0.01 *vs* WKY; ^#^P<0.05 and ^##^P<0.01 *vs* SHR (one-way ANOVA).

The body weight of rats was not affected by captopril administration (data not shown), while systolic blood pressure was significantly decreased by captopril treatment (Figure 1).

### Effect of captopril on renal function

**Figure 2.**
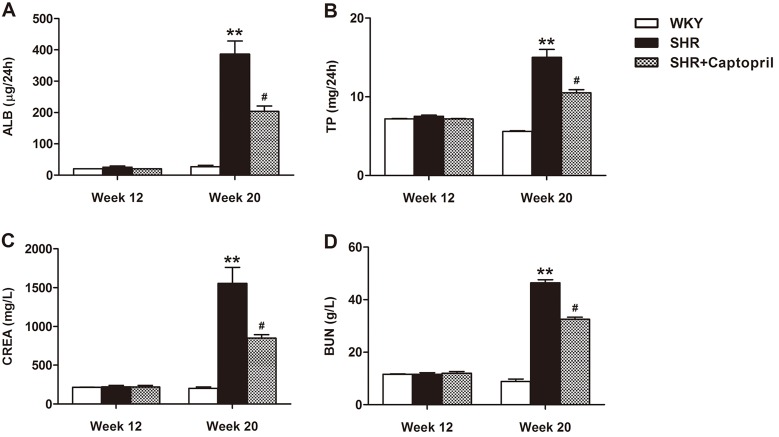
Treatment of spontaneously hypertensive rats (SHR) with captopril improved renal function compared with Wistar-Kyoto (WKY) rats. Urinary albumin (ALB), total protein (TP), serum creatinine (CREA), and blood urea nitrogen (BUN) were measured using specific kits. Data are reported as means±SE (n=6 in each group). **P<0.01 *vs* WKY; ^#^P<0.05 *vs* SHR (one-way ANOVA).

Renal function was assessed by urinary ALB, TP, serum CREA, and BUN analysis (Figure 2). At week 12, these biochemical parameters in the SHR group did not change significantly compared to the WKY group. However, the levels of ALB, TP, CREA, and BUN in SHR were significantly higher than those in WKY rats (P<0.01), indicating the obvious renal injury in 20-week SHR rats, which could be partially inhibited by captopril treatment for 13 weeks (P<0.05).

### Effect of captopril on renal histology

**Figure 3.**
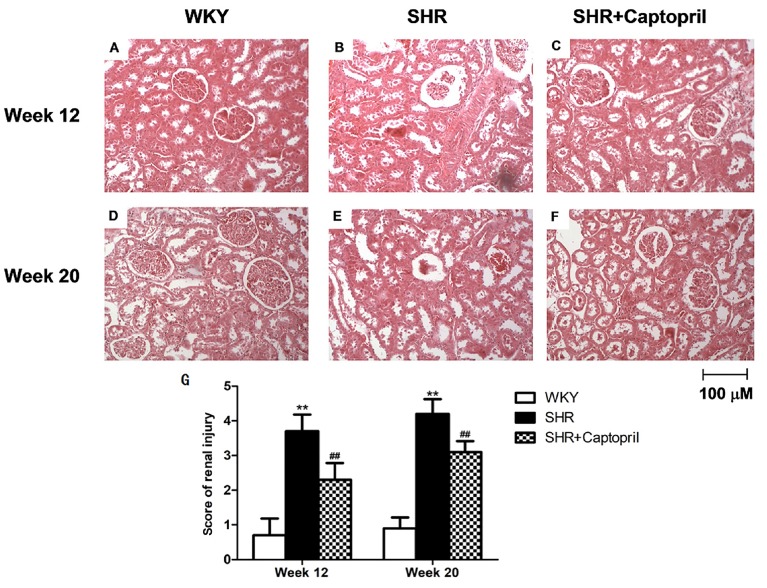
Treatment of spontaneously hypertensive rats (SHR) with captopril improved renal histology. *A*, *D*, Wistar-Kyoto rats (WKY) at week 12 and week 20 showing no abnormal changes. *B*, SHR at week 12 with presence of minimal glomerular atrophy, fall-off of tubule epithelial cells, interstitial fibrosis, and increased thickness of basement membrane. *E*, SHR at week 20 showing significant pathological changes in the kidney. *C*, *F*, SHR+Captopril at week 12 and week 20 showing improvement of kidney histology. Magnification: 200×; bar: 100 μm. *G*, Renal injury scores (n=6 in each group). Data are reported as means±SE. **P<0.01 *vs* WKY; ^##^P<0.01 *vs* SHR (one-way ANOVA).

As shown in Figure 3, histopathological examination showed no obvious abnormality in the kidney structures in the WKY group. However, at week 12, SHR showed tubular cell necrosis, exfoliated cells in the lumen, and vascular congestion obviously increased, which was aggravated at week 20. The scores of renal changes were 0.70±0.48 and 0.90±0.32 in the WKY group at week 12 and 20. The scores of renal changes were 3.70±0.48 and 4.20±0.42 in the SHR group at week 12 and 20 (P<0.01). However, these pathological changes were partially prevented by captopril treatment at week 12 and 20 (P<0.01) (Figure 3).

### Effect of captopril on expression of inflammatory factors

**Figure 4.**
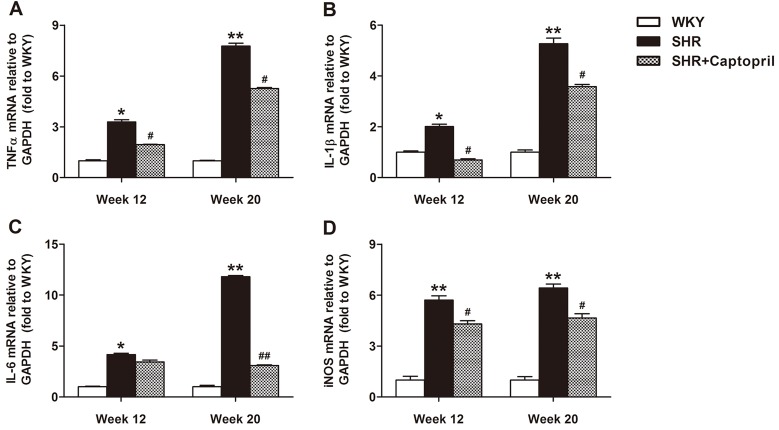
mRNA expression of inflammatory markers tumor necrosis factor α (TNFα), interleukin (IL)-1β, IL-6, and induced nitric oxide synthase (iNOS) in spontaneously hypertensive (SHR) and Wistar-Kyoto (WKY) rats. Data were obtained by real-time PCR and the results are reported as means±SE relative to GAPDH (n=6 in each group). *P<0.05 and **P<0.01 *vs* WKY; ^#^P<0.05 and ^##^P<0.01 *vs* SHR (one-way ANOVA).

**Figure 5.**
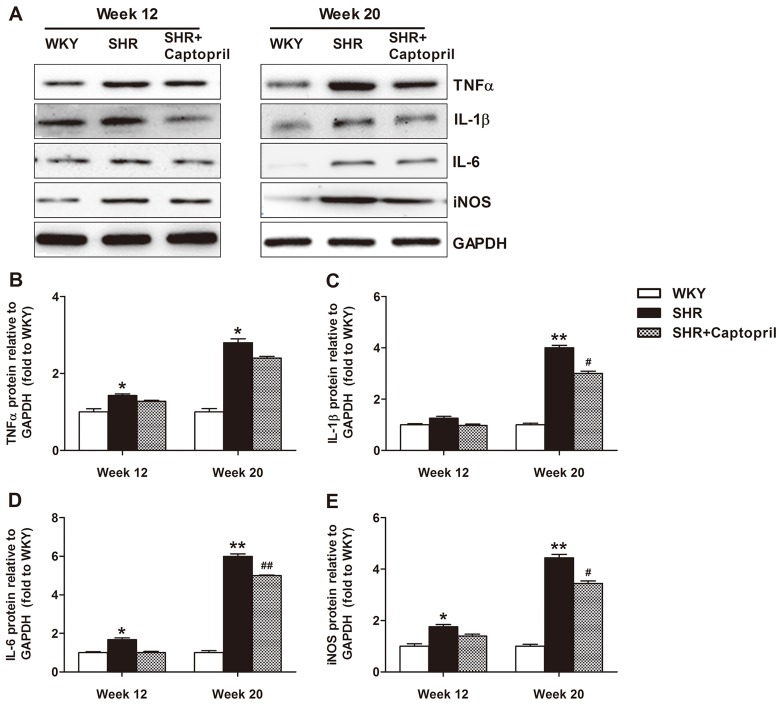
Protein expression of tumor necrosis factor-α (TNFα), interleukin (IL)-1β, IL-6, and induced nitric oxide synthase (iNOS) in spontaneously hypertensive (SHR) and Wistar-Kyoto (WKY) rats. Data were obtained by western blot and are reported as means±SE relative to GAPDH (n=6 in each group). *P<0.05 and **P<0.01 *vs* WKY; ^#^P<0.05 and ^##^P<0.01 *vs* SHR (one-way ANOVA).

We further investigated if inflammation was involved in the renal protective effect of captopril in SHR rats. As shown in Figure 4, the inflammatory factors including TNF-α, IL-1β, IL-6, and iNOS mRNA levels in SHR rats were increased at week 12, and further enhanced at week 20, compared to the WKY group. This change could be partially repressed by captopril treatment. In addition, the protein expression of these inflammatory cytokines exhibited a similar pattern with mRNA levels (Figure 5).

### Effect of captopril on NF-κB activation

**Figure 6.**
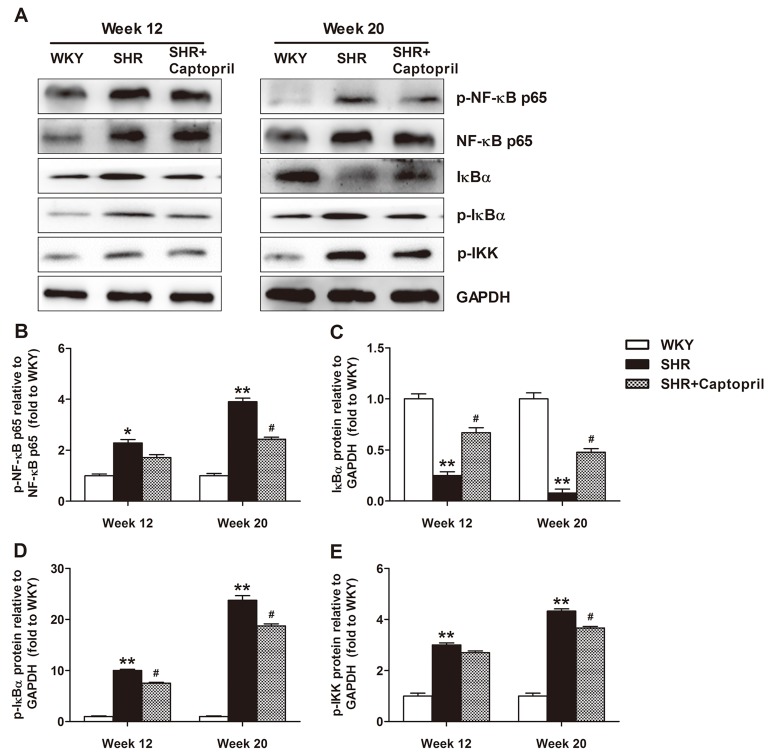
Nuclear factor-κB (NF-κB) activation in spontaneously hypertensive (SHR) and Wistar-Kyoto (WKY) rats. *A*, Typical traces were representative of six experiments with similar results. *B-E*, Data are reported as means±SE relative to GAPDH (n=6 in each group). *P<0.05 and **P<0.01 *vs* WKY; ^#^P<0.05 *vs* SHR (one-way ANOVA).

Phosphorylation levels of NF-κB p65, IκBα, and IKK in SHR rats were higher, while the expression of IκBα was significantly lower than that in WKY rats at week 12. This tendency became more evident at week 24, indicating enhanced NF-κB activation along with the progress of hypertension. Captopril treatment partially ameliorated the NF-κB activation in the kidneys of SHR rats (Figure 6).

### Effect of captopril on endothelium-dependent vasorelaxation

**Figure 7.**
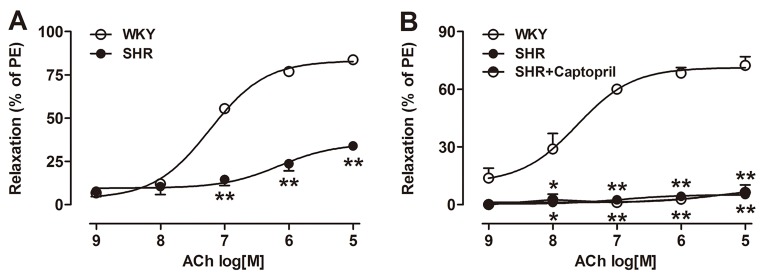
Cumulative concentration-response curves to acetylcholine (ACh) in endothelium-intact renal arteries from spontaneously hypertensive (SHR) and Wistar-Kyoto (WKY) rats at week 12 (*A*) and week 20 (*B*). The dilation responses to ACh are reported as the percentage of contractile response induced by 1 µM phenylephrine (PE). Each point represents means±SE (n=6). *P<0.05 and **P<0.01 *vs* WKY (one-way ANOVA).

As shown in Figure 7, the endothelium-dependent relaxation to ACh in SHR rats decreased significantly compared with WKY rats at week 12, and the decrease was aggravated at week 20, indicating the progressive endothelial dysfunction of the renal artery during the progress of hypertension. It is worthy to note that renal artery endothelial dysfunction in SHR rats was unchanged after 13 weeks of captopril treatment, suggesting that the endothelial function might not be involved in the renoprotective effects of captopril on SHR rats.

## Discussion

The present study showed that the renal damage in SHR rats would be aggravated gradually along with the progress of hypertension, and captopril provided significant renal protective effects on SHR rats. However, captopril did not ameliorate the renal artery endothelial dysfunction in SHR rats.

Accumulating evidence has demonstrated that inflammation plays an important role in the pathogenesis or renal injury (10,11), and anti-inflammation drugs have been shown to have potential renoprotective effects in hypertension (12,13). In accordance with previous studies, captopril improved abnormality in rat kidney structures at week 12 and 20. Early studies have shown that the expression of pro-inflammatory cytokines such as TNF-α (14,15), IL-1β (16,17), and IL-6 (13,18) in kidneys are elevated and contribute to local tissue injury in hypertension. In addition, iNOS is a major inflammatory mediator that contributes to the pathogenesis of renal inflammation (19). In the present study, 12-week-old SHR exhibited higher expression of inflammatory factors including TNF-α, IL-1β, IL-6, and iNOS at both mRNA and protein levels, which could be aggravated with the progress of hypertension and attenuated by captopril treatment. The results clearly demonstrated that captopril treatment could attenuate renal inflammation. However, the present study cannot determine whether the anti-inflammatory effects observed in the SHR-captopril group was a result of a direct effect of captopril or just secondary changes simply due to the reduction of blood pressure.

Additionally, numerous studies have shown that the NF-κB pathway contributes to transcriptional upregulation of inflammatory factors in the pathogenesis of renal dysfunction (20–23). Activation of the NF-κB is initiated by the signal-induced degradation of IκB proteins (24), which occurs primarily via activation of a kinase called IκB kinase (IKK) (25). With the degradation of IκB, the NF-κB complex is then freed to enter the nucleus where it can induce the expression of specific genes that have DNA-binding sites for NF-κB nearby (26,27). Our present study found that SHR exhibited higher phosphorylation levels of NF-κB, IκBα, and IKK, and lower IκBα expression in kidneys compared with WKY at both week 12 and week 20 (Figure 6). The activation of NF-κB signaling might contribute to the augmented expression of inflammatory factors in SHR kidneys. Figure 6 also showed that this change could be suppressed by captopril treatment, indicating that inflammatory factor regulation via NF-κB activation is possibly involved in the renoprotective effect of captopril on SHR rats.

It has been demonstrated that endothelium-dependent relaxation is attenuated in hypertension (a phenomenon referred to as endothelial dysfunction) and contributes to the increase of peripheral resistance (28,29). The impaired vasodilator response is a risk factor for renal function loss in patients with essential hypertension (30). In the present study, the endothelium-dependent vasorelaxation in SHR was impaired at week 12 and aggravated at week 20, with approximately 95% damage. However, this change could not be ameliorated by captopril treatment, suggesting that endothelial function was not involved in the renoprotective effect of captopril on SHR rats.

It is worthy to note that systolic blood pressure was higher in SHR compared with WKY at week 7, while the markers of renal damage including ALB, TP, CREA, and BUN did not exhibit significant alteration at week 12 but did at week 20. However, NF-κB activation, renal inflammation, and morphology change were significantly present in SHR at week 12, suggesting that new early biomarkers for renal damage instead of ALB, TP, CREA, or BUN are needed.

In conclusion, captopril improved renal damage and inflammation in SHR, which might be due to its inhibiting NF-κB activation. Although renal endothelial dysfunction was unchanged by captopril treatment, it is possible that better renal protection would be achieved by using captopril and other agents for improving endothelial function.
